# Reactive hyperplastic lesions of the oral cavity: A ten year observational study on North Indian Population

**DOI:** 10.4317/jced.50670

**Published:** 2012-07-01

**Authors:** Vandana Reddy, Susmita Saxena, Sanjeev Saxena, Munish Reddy

**Affiliations:** 1 Lecturer, Department of Oral Pathology and Microbiology, Subharti Dental College, Meerut, India.; 2 Professor and Head, Department of Oral Pathology and Microbiology, Subharti Dental College, Meerut, India.; 3 Professor and Head, Department of Oral and Maxillofacial surgery, Subharti Dental College, Meerut, India.; 4 Professor, Department of Orthodontics, Subharti Dental College, Meerut, India.

## Abstract

Back ground: The aim of this study was to determine the frequency of focal reactive hyperplastic lesions of the oral cavity as reported in the Department of Oral Pathology and Microbiology, Subharti Dental College, Meerut and to compare these data with those of previously reported studies from other regions and countries. Material and Method: Patient records of the Department of Oral Pathology were retrieved during a 10 year period from 2001 to 2010. Data of all reactive hyperplasias namely focal fibrous hyperplasia (FFH), pyogenic granuloma (PG), peripheral ossifying fibroma (POF) and peripheral giant cell granuloma (PGCG) were reviewed and analyzed for age, gender, and site of location. Results: There were 209 focal reactive hyperplastic lesions that comprised 12.8% of the 1634 accessed biopsies. FFH was the most common lesion constituting 57.4% of the cases, followed by PG (18.7%), POF (17.7%) and PGCG (6.22%). The mean age of patients at presentation was 31.56 years. The female to male ratio was 1.5:1. The most frequently involved site was the gingiva (81.8%); other sites were the buccal mucosa, lips, tongue, alveolar mucosa and palate. Conclusion: Oral lesions are often detected by Dental professionals and surgeons. Knowledge of the frequency and presentation of the most common oral lesions is beneficial in developing a clinical impression of such lesions encountered in practice and to minimize potential dentoalveolar complications.

** Key words:**Focal reactive hyperplastic lesions, fibrous hyperplasia, pyogenic granuloma, peripheral ossifying fibroma, peripheral giant cell granuloma.

## Introduction

Oral mucosa is constantly subjected to external and internal stimuli and therefore manifests a spectrum of diseases that range from developmental, reactive and inflammatory to neoplastic (1). Reactive hyperplastic lesions represent the most frequently encountered oral mucosal lesions in humans (2). These lesions represent a reaction to some kind of irritation or low grade injury like chewing, trapped food, calculus, fractured teeth and iatrogenic factors including overextended flanges of dentures and overhanging dental restorations (3). Kfir et al (1980) have specifically classified reactive hyperplastic lesions into pyogenic granuloma (PG), peripheral giant cell granuloma (PGCG), peripheral ossifying fibroma (POF) and fibrous hyperplasia (FH) (4). Not much difference exists in clinical appearance among various hyperplastic lesions. As a result Periodontologists and Oral and Maxillofacial Surgeons often give the diagnostic term ‘epulis’ to these lesions clinically (5). Diagnosis of each lesion from this subgroup is aided by their clinical and radiographic features but histopathology is the key for final diagnosis (6). Most data about reactive hyperplastic lesions of the oral cavity come from Western countries and despite a considerable volume of publications, reactive hyperplasia has not as yet been studied in the Indian population. The aim of this study is to analyze the clinicopathological features of the cases diagnosed as hyperplastic reactive lesions of the oral cavity from Department of Oral Pathology and Microbiology, Subharti Dental College, Meerut during a 10 year period and to compare the results with the reported data in the scientific literature.

## Material and Methods

**Figure 1:**
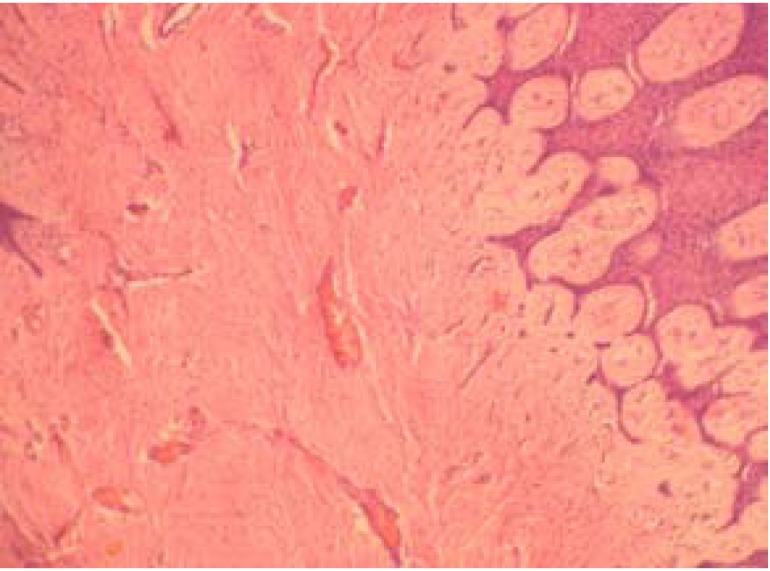
Fibrous hyperplasia showing hyperplastic epithelium with bundles of collagen fibers (H & E X100).

**Figure 2:**
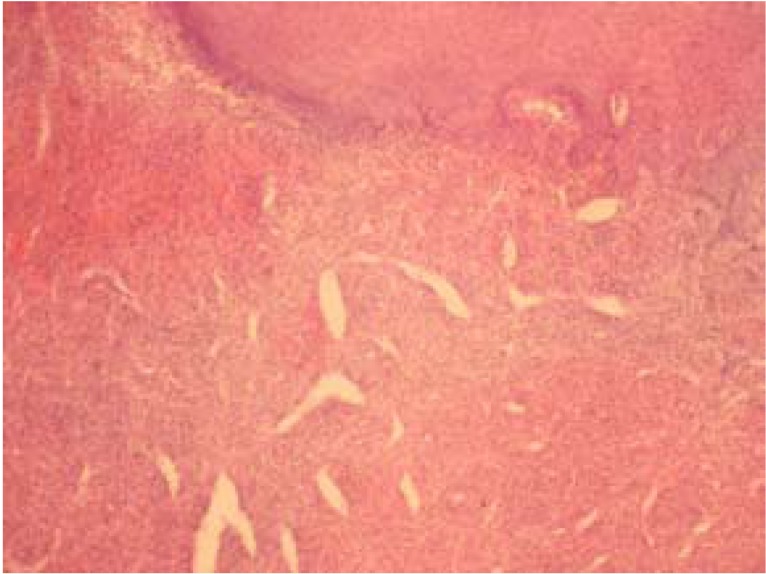
Pyogenic granuloma with hyperplastic epithelium that overlies a fibrous connective tissue that contains numerous chronic inflammatory cells and blood vessels (H & E X100).

**Figure 3:**
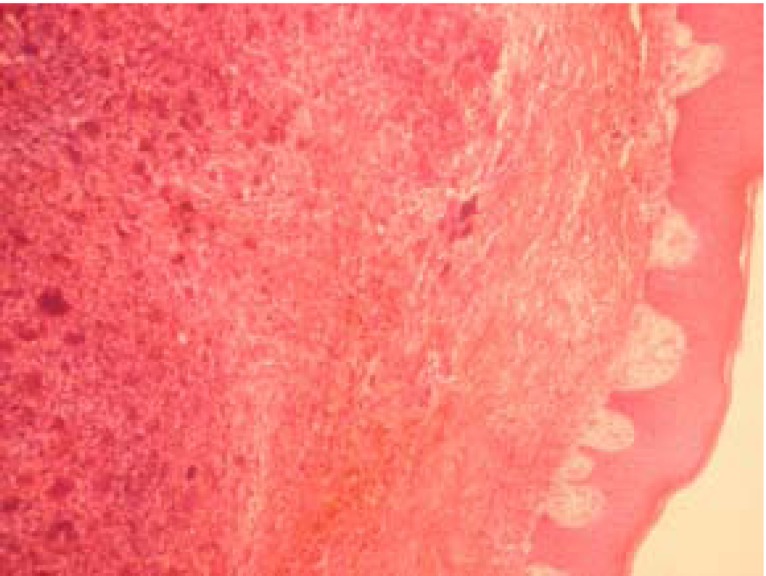
Peripheral ossifying fibroma with fibrous connective tissue containing calcified deposits (H & E X100).

**Figure 4:**
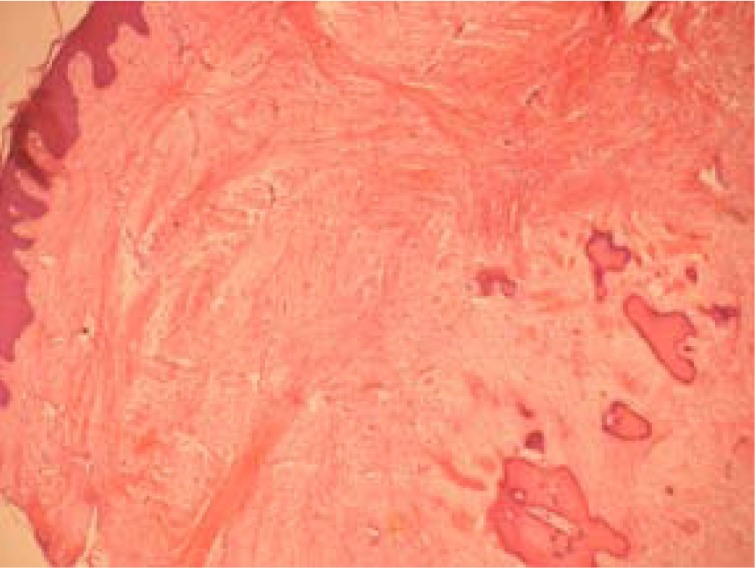
Peripheral giant cell granuloma with multinucleated giant cells, extravasated RBC’s and deposits of hemosiderin (H & E X100).

In this retrospective study all the existing records in the archives of Oral Pathology and Microbiology, Subharti Dental College, Meerut were extracted between 2001 and 2010. Patient records were assessed to select those with the histopathological diagnosis of reactive hyperplastic lesions as classified by Kfir et al (1980) (4). The cases for inclusion in this study were those categorized as fibrous hyperplasia, pyogenic granuloma, peripheral ossifying fibroma and peripheral giant cell granuloma (Figs. 1,2,3,4). Clinical data regarding age, gender, location of the lesion were obtained for each case from the patient records. Descriptive statistical methods (mean, standard deviation and percent) were applied to data and z-test was employed to assess mean differences.

## Results

From a total of 1634 records evaluated during 10 year interval 209 of the lesions were reactive hyperplasia. This constituted 12.8% of the total biopsies accessed during the period. The most common lesion was found to be fibrous hyperplasia with 120 cases (57.4%).Followed by 39 cases (18.7%) of pyogenic granuloma, 37 cases (17.7%) of peripheral ossifying fibroma and 13 cases (6.22%) of peripheral giant cell granuloma. Of all the patients examined 84 were males and125 were females and the ratio was 1:1.5. The age of patients ranged from 7 to 82 years with a mean age of 31.56 years. The mean age of patients with focal fibrous hyperplasia, pyogenic granuloma, peripheral cemento-ossifying fibroma and peripheral giant cell granuloma was 36.56, 28.04, 32.49 and 29.16 years respectively ( Table 1). No statistical significant difference in mean age was observed between the two genders (p<0.01). Gingiva was the most common site with 171 cases (81.8%) followed by buccal muco-sa with 17 cases (8.1%), lip with 7 cases (3.35%), palate with 6 cases (2.9%), tongue with 5 cases (2.4%) and alveolar mucosa with 3 cases (1.43%) ( Table 2). By applying one way Annova significant difference amongst different sites was observed in different lesions at 1% level of significance (p<0.01) (T Table 3, Table 4).

**Table 1:**
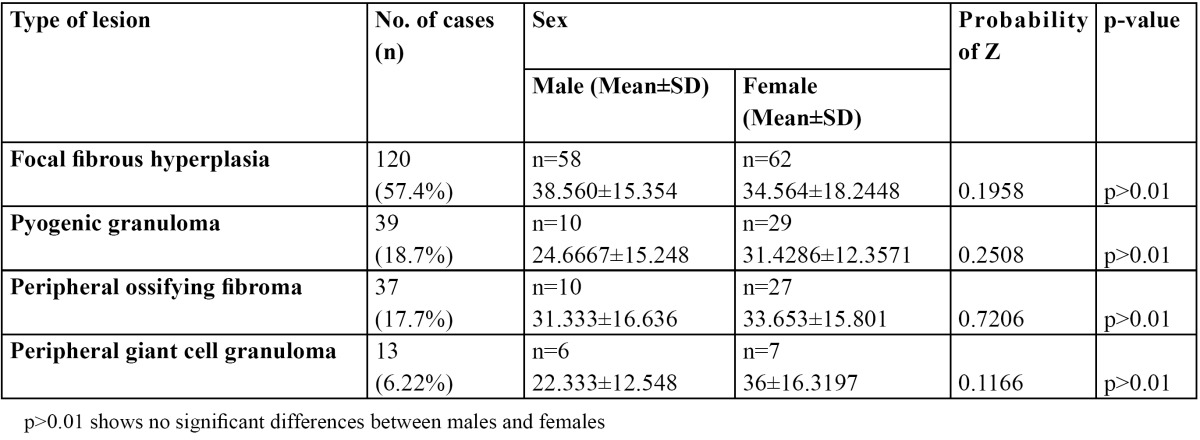
Distribution of 209 lesions of reactive hyperplasia according to sex, age range and mean age.

**Table 2:**
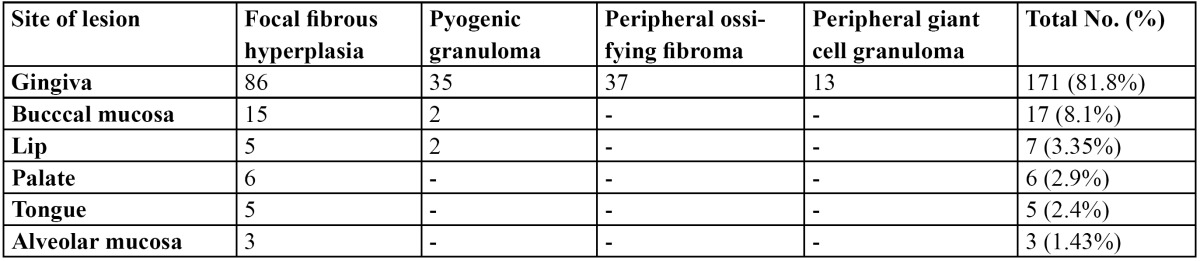
Distribution of 209 lesions of reactive hyperplasia according to site of location.

**Table 3:**
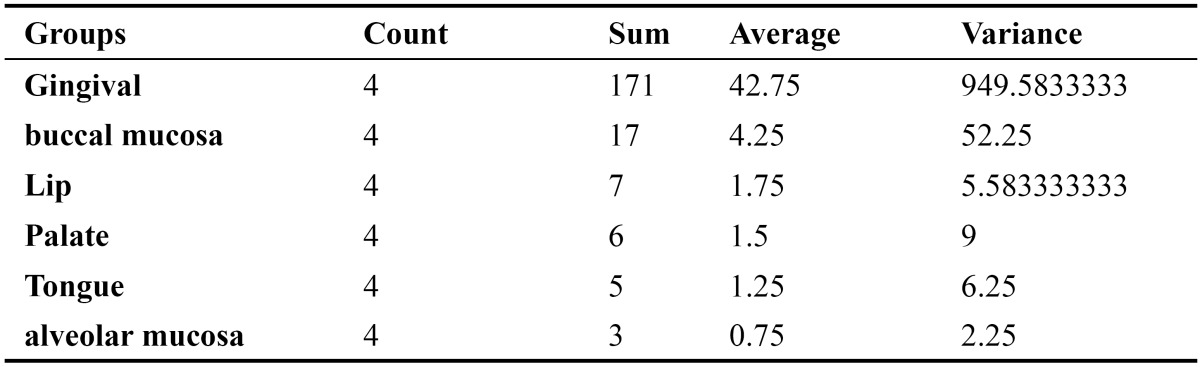
Distribution of 209 lesions of reactive hyperplasia according to site of location.

**Table 4:**
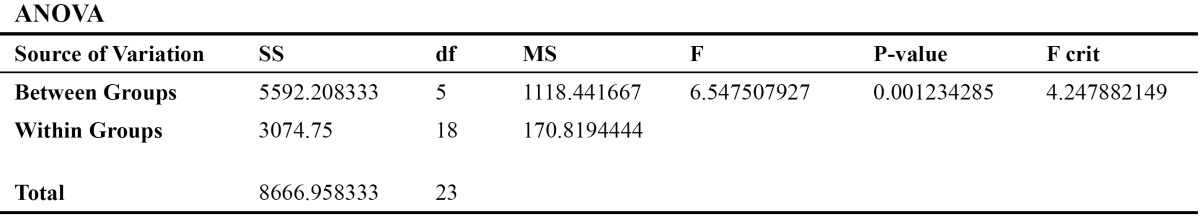
Source of variation of distribution of 209 lesions of reactive hyperplasia according to site of location.

## Discussion

Reactive hyperplasia comprises of a group of fibrous connective tissue lesions that commonly occur in the oral mucosa as a result of injury or chronic irritation (7). Chronic trauma can induce inflammation which produce granulation tissue with endothelial cells, chronic inflammatory cells and later fibroblasts proliferate and manifest as an overgrowth called Reactive hyperplasia. These tumor like lesions are not neoplastic, but they indicate a chronic process in which an exaggerated repair occurs (granulation tissue and formation of scar) following repair (8). They are categorized into four subgroups which includes pyogenic granuloma, peripheral ossifying fibroma, peripheral giant cell granuloma and fibrous hyperplasia (Kfir et al) (4). Local reactive hyperplastic lesions of the oral cavity are relatively common in biopsy services of Oral Pathology (9) They comprised 12.4% of the 1634 biopsies we accessed in this study. These observations are in support with studies by Awange et al and Nartey et al which comprised of 10.6% and 10.3% respectively (10,2).

It is interesting that most reactive hyperplastic lesions occurred in the female gender with female to male ratio of 1.5:1. In a study carried out by Zarei et al and Aghbali et al they were more common in females (male to female ration of 1:1.8 and 1: 1.4) (3,11). In this series, there was a high degree of occurrence of reactive hyperplastic lesions of the oral cavity in 2nd, 3rd and 4th decades of life. This age distribution is in accordance with age distribution reported in previous studies (11). The present series has also shown that the mean age of occurrence of these lesions is 31.56 years. We found that the principal oral site affected is the gingiva. Other oral sites in the descending order of occurrence are buccal mucosa, palate, lip and tongue (Aghbali et al) (11).

Fibrous hyperplasia accounts for the great majority of localized reactive lesions as was substantiated by various reports in the literature (9). It comprised of 57.4% of all the reactive lesions and 7.34% of total number of biopsies accessed. Although the fibrous hyperplasia can occur anywhere in the mouth, the most common location is the gingiva followed by bucccal mucosa with 41.1% and 7.2% respectively (3). These lesions are most common in the 2nd to 4th decades of life and the mean age of occurrence of the lesion is 36.56 years (4). These lesions are seen slightly more in the females with female to male ratio of 1.1:1 (12).

Pyogenic granuloma was the second most common lesion comprising of 18.7% of all the reactive lesions and 2.39% of the total number of biopsies accessed (13). We found that the principal site affected by pyogenic granuloma was the gingiva. Other oral sites were the lower lip, tongue, buccal mucosa and palate. These findings are consistent with those of others (14). Our findings with regard to gender also confirm the previous conclusion that pyogenic granuloma is more common in females with female to male ratio of 2.9:1 (15,16). In our study PG most frequently occurred in the third to fourth decade which was similar to those reported by Buchner et al where the mean age of occurrence was 28.04 years (9,14).

Peripheral ossifying fibroma was the third most common lesion comprising of 17.7% of all the reactive lesions and 2.26% of total biopsies accessed (6). The male to female ratio is 1:2.7 suggesting a female predilection which was consistent with the results reported by Zhang et al and Kfir et al (4,5). Our results also showed the peak incidence in the third decade and the mean age was 32.49 years supported by Kfir et al and contrasted with the findings of Zhang et al (4,5).

The peripheral giant cell granuloma was the least common lesion in this study comprising of 6.22% of all the reactive lesions and 0.8% of all the biopsies accessed (17). In this study the age of patient ranged from 12-60 years with mean age of 29.16 years and with the highest incidence in the second decade of life similar to other studies (4). PGCG affects females more than males with a proportion of1.2:1 ( Reichart and Philipsen with1.5:1) (18).

This study supports previous assertion that fibrous hyperplasia and PG may occur on any oral mucosal site with special preference for the gingiva while PGCG and POF occur exclusively on the gingiva (2). Eversole and Rovin suggested that limitations of PGCG and POF to the gingiva supports a possible histogenic derivation from the superficial periodontal ligament which contain cells capable of producing bone and cementum (19).

The finding that the majority of lesions affected female patients could reflect a greater concern and compliance in female patients towards dental care or the role of hormones. The lower number of lesions biopsied from older patients, i.e in the age groups of 71 to 80, most likely reflects the fact that people at this age are often edentulous. In addition, people in these age groups do not usually receive regular dental check ups and most of their painless lesions remain undiagnosed (20).

The frequent gingival site of occurrence supports an assertion that these hyperplastic lesions are the same lesions at different developmental stages. Daley et al suggest the vascular component of PG is gradually replaced by fibrous tissue with time and hence diagnosed as a fibrous hyperplasia or fibroma (12). On the contrary our study did not show a clear cut age grouping for the various entities. The mean ages for the different lesions was not shown to distinctly reflect the progressive development of the lesions through the different histological stages, therefore whether or not the focal reactive lesions represent the same lesion at different developmental stages is questionable. Eversole and Rovin speculated that the different histological entities of inflammatory hyperplasia may be due to connective tissue response to varied intensities of mucosal irritation (16).

## Conclusion

We are of the opinion that FH, PG, PGCG and POF are mucosal responses to chronic low grade irritation caused by plaque, and calculus or any other irritant. However, histologic appearance of each entity may be influenced by the intensity of irritation, duration of the lesion and possibly the effects of hormones. It is helpful to know the frequency and presentation of the most common oral lesions in order to develop a clinical impression of such lesions met in practice. Identification of any reactive hyperplastic lesion requires the formulation of a differential diagnosis to enable accurate patient evaluation and management. So, early diagnosis and removal of these lesions along with removal of the irritant can greatly minimize potential dentoalveolar complications.
